# Verbal Shadowing and Visual Interference in Spatial Memory

**DOI:** 10.1371/journal.pone.0074177

**Published:** 2013-09-03

**Authors:** Tobias Meilinger, Heinrich H. Bülthoff

**Affiliations:** 1 Max Planck Institute for Biological Cybernetics, Tübingen, Germany; 2 Research Center for Advanced Science and Technology, the University of Tokyo, Tokyo, Japan; 3 Department of Brain and Cognitive Engineering, Korea University, Seoul, Korea; Utrecht University, Netherlands

## Abstract

Spatial memory is thought to be organized along experienced views and allocentric reference axes. Memory access from different perspectives typically yields V-patterns for egocentric encoding (monotonic decline in performance along with the angular deviation from the experienced perspectives) and W-patterns for axes encoding (better performance along parallel and orthogonal perspectives than along oblique perspectives). We showed that learning an object array with a verbal secondary task reduced W-patterns compared with learning without verbal shadowing. This suggests that axes encoding happened in a verbal format; for example, by rows and columns. Alternatively, general cognitive load from the secondary task prevented memorizing relative to a spatial axis. Independent of encoding, pointing with a surrounding room visible yielded stronger W-patterns compared with pointing with no room visible. This suggests that the visible room geometry interfered with the memorized room geometry. With verbal shadowing and without visual interference only V-patterns remained; otherwise, V- and W-patterns were combined. Verbal encoding and visual interference explain when W-patterns can be expected alongside V-patterns and thus can help in resolving different performance patterns in a wide range of experiments.

## Introduction

Overwhelming evidence indicates that spatial memory is orientation dependent: accessing spatial knowledge from certain perspectives is faster and/or more accurate than accessing it from other perspectives [1]. However, results differ in the number of perspectives participants perform better in, as indicated by V- and W-shaped performance patterns.

**Figure 1:**
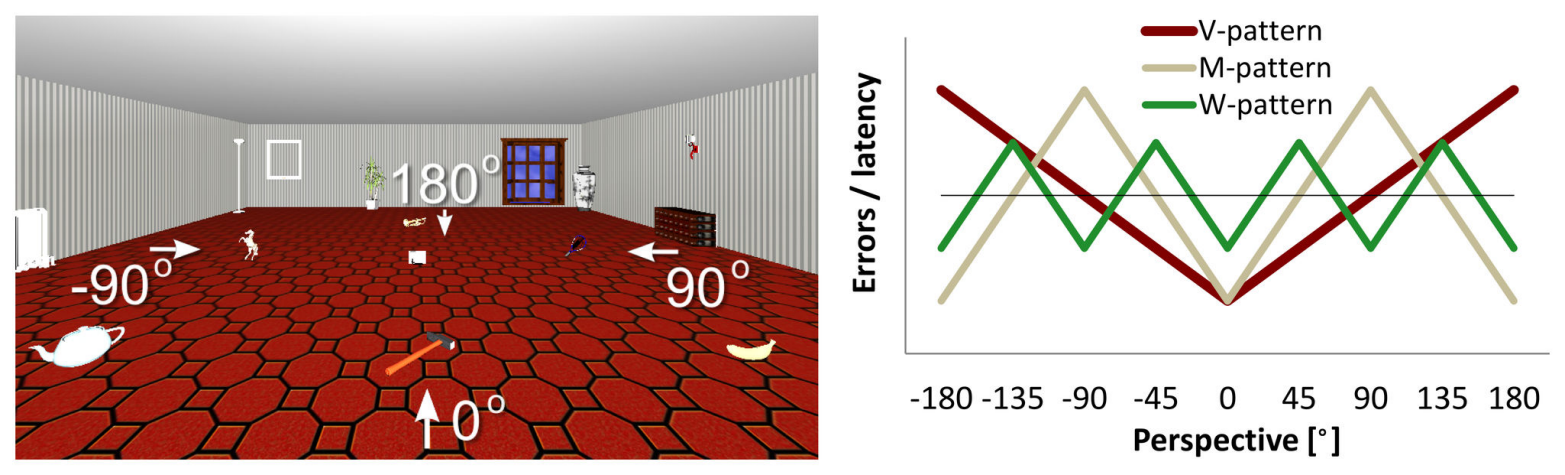
Spatial memory access from different perspectives. Left: Virtual object array as seen by participants in the experiment. Main axes are indicated in addition. Right: Performance in spatial memory access as predicted by V-, W-, and M-patterns. Data for −180° are displayed twice for symmetry.

**V-shaped** patterns typically show the best performance when tested from an experienced perspective; errors and latencies increase monotonically with the angular deviation from this perspective—not necessarily linearly (Figure 1). V-shapes have been observed in recognizing objects or scenes [2–4] or in indicating one’s current perspective in maps relative to buildings seen before [5]. Matching two objects displayed in different perspectives [6] or pointing and configuration judgments based on map-acquired knowledge also result in V-patterns [7–9]. Typically, V-patterns are centered on egocentrically experienced orientations. Multiple V-patterns may combine with each other [10]. Often contra-aligned test perspectives (i.e., 180°) yield comparatively better performance than misalignments of 135° [2,8,9]. Contra-alignment might allow for different retrieval processes (e.g., mirroring, exchange left/right). Other misalignments that require mental rotation or that affect similarity estimates between the presented and encoded view may lead to monotonic increases in error/latency across perspectives.

**W-shape**, or sawtooth, patterns have been observed when participants learned object arrays and conducted judgments of relative directions afterwards, i.e., they imagined standing at one object in the array, facing another object, and indicated the direction of a third object [3,4,11–16]. W-patterns have also been obtained in judgments on university campus locations [17] and after walking in a rectangle around a temple [18]. Usually, the W-pattern is considered allocentric; it is aligned with orientations intrinsic to the layout and/or the surrounding room (i.e., parallel/orthogonal to the main axes) even when all or some encountered perspectives are not. W-patterns are thought to originate from encoding spatial information relative to one or two orthogonal reference axes of a reference frame. Imagined perspectives from along the endpoints of these axes are retrieved, whereas other perspectives have to be inferred, involving costs in latency and errors [1,19].

It is puzzling that some tasks predominantly yield V-patterns presumably relying on egocentric views (i.e., recognition, visual comparison, and visual pointing and self-localizing after map learning), while other tasks seem to foster allocentric axis encoding with subsequent W-patterns (i.e., imagined pointing, mainly after learning object arrays). The present study examined how experimental circumstances yield W-patterns rather than V-patterns. We proposed and tested the hypotheses that W-patterns are a result of verbal encoding as well as visual interference during retrieval.

### The verbal encoding hypotheses

The orientations along which a layout is described and along which participants perform best during pointing are closely related. When participants indicate reference objects in a previously seen object array (e.g., A is next to object X), they preferentially select objects located along axes which also yield better pointing performance [20]. In describing an array, participants showed W-shaped pointing patterns centered on the described perspective rather than centered on the perspective from which they previously viewed the array [4]. Verbal descriptions and orientations of best pointing thus coincided. According to the verbal encoding hypothesis, this happens because inter-object relations are also encoded in a verbal format, which is then used in subsequent pointing. Object arrays typically applied are easily described row by row or column by column. If memorized in such a descriptive format, retrieving locations will be easier along rows or columns as compared with oblique orientations based on both rows and columns. This will yield better pointing along columns and rows and thus to the W-patterns observed. Descriptions do not necessarily have to be “teapot, hammer, banana, etc.”; other forms are acceptable, such as “the teapot is left of the hammer, the horse is behind the teapot, etc.” The crucial point is that the description is verbal and that it is organized along rows and columns.

Descriptions along rows and columns are a specific form of encoding relative to reference axes. These descriptions are relative to other objects. If array orientation deviates from the observed perspective, description may be along array orientations. Subsequent pointing based on these descriptions will be better along array orientations and not experienced orientations [4,12]. The advantage of specifying axes encoding as verbal is that it predicts *when* axes encoding and subsequent W-patterns will be observed, namely when verbal codes are used. Experiments in which V-patterns were observed often did not make verbal memory codes easier: visual comparisons do not require memory [6] and map layouts might be learned mainly in a visuospatial format [7–9]. Recognition performance was even shown to decline when the stimuli were recoded verbally [21], and scene recognition typically shows V-shaped patterns [2–4].

In order to test the verbal encoding hypothesis, participants learned an object layout either with or without verbal shadowing. Verbal shadowing inhibits the formation of a verbal memory trace [22]. According to the verbal encoding hypothesis W-patterns are based on verbal memory traces. Therefore, inhibiting verbal encoding by verbal shadowing should also reduce W-patterns in subsequent pointing relative to the control condition without shadowing. Verbal encoding is one process proposed to yield W-patterns when learning object arrays. However, also visual interference might result in W-patterns.

### The visual interference hypotheses

Reasoning about locations within a room from an imagined position different than one’s current body position yields sensorimotor interference between the de facto and the imagined surroundings [19,23–26]. The reader may experience this by imagining turning 90° to the left and then pointing to this text from the imagined perspective. Often a strong tendency to point from the physical (i.e., to the front) rather than the imagined perspective (to the right) is experienced.

This interference typically increases with the turning angle between de facto and imagined surrounding [24,25]. However, in addition to the effect of turning angle, better performance was observed when the de facto and imagined positions were both aligned with the surrounding walls compared with when the imagined position was not [24]. Wall misalignment might thus yield stronger interference than wall alignment. We now propose interference to happen not only when imagining a different position within the same environment, but also when retrieving previously learned information between the currently visible surrounding and the recalled environment (see 27 for an initial support of this assumption). Since the room geometry is learned quickly [28], such a cue will be available during recall and can thus interfere with the geometry of the room currently located within. This visual interference might generate a W-shaped pattern in a typical imagined pointing task, in which participants sit in a test room presumably aligned with the walls of the test room and imagine standing in the learning room in different perspectives. When imagining perspectives parallel to walls (i.e., 0°, ±90°, 180°), imagined and physical walls will be parallel. Now one could imagine the walls of physical room being the walls of the imagined room. This should not cause much interference. However, when imagining oblique perspectives, imagined room corners will be along the walls of the physical room and vice versa. This will cause comparatively larger interference and thus higher errors/latency to occur in imagined oblique perspectives, ergo a W-pattern. The idea is that a visible room and a recalled room will interfere with each other the less the more similar they are, i.e., the more they match when mentally superimposed. To test this hypothesis, participants learned an object array within a room while aligned with the walls. Afterwards they pointed within this room with the room either being visible or not. Visual interference yielding a W-pattern requires a visible room surrounding. Therefore, we expected larger W-patterns with the surrounding room visible compared with no room visible.

Visual interference happens during retrieval, verbal encoding during encoding. We expected both effects to independently influence W-patterns. In order to investigate independence, both predictions were tested in parallel, resulting in a crossed 2 (verbal shadowing yes/no) × 2 (visual interference yes/no) experimental design; we predicted no interaction between the factors. Furthermore, if verbal encoding and visual interference are the driving forces behind W-patterns, then W-patterns should largely vanish in the condition with verbal shadowing and without interference.

As a control, we also examined an M shaped pattern whose spatial frequency is right in between V and W patterns as shown in Figure 1: from 360° of possible test perspectives, a V pattern predicts best performance at one perspective (0°), an M pattern at two perspectives (0° and 180°) and a W pattern at four perspectives (0°, +90°, -90°, and 180°). Performance is thought to decrease in between these best perspectives. The often observed relative better performance at contra-aligned perspectives [2,8,9] contributes to an M-pattern and will thus be tested.

The present study’s aim was to examine whether W patterns in spatial memory access originate from verbal encoding and visual interference, whether these effects are independent from each other, and whether they are the main sources for W-patterns. We observed support for all three predictions.

## Methods

### Participants

Thirty-two naïve participants (13 women), aged 18 to 44 years (*M* = 27.3; *SD* = 5.5) were recruited from a subject database and participated in exchange for monetary compensation. All participants were German native speakers or spoke German on a comparable level.

### Ethics statement

The study was approved by the ethics committee of the University Clinic, Tübingen. All participants gave written informed consent before conducting the experiment.

### Materials

Participants saw a virtual room containing seven target objects lying on the floor, through a head mounted display (HMD), while standing at a table with a mounted joystick (Figure 1). The array layout used in many experiments [11,14,29,30] consisted of an incomplete 3 × 3 grid with bilateral symmetry. The closest row contained a teapot, a hammer, and a banana, and the middle row held a horse, a telephone, and a tennis racket. A trumpet was located in the center of the furthest row. Additional objects by or on the walls indicated the orientation of the rectangular room. Learning perspective, intrinsic object layout, and room orientation all predicted selection of the same reference system (0° perspective).

The experiment was programmed in Virtools® 5.0 (Dassault Systemes). Participants’ head coordinates were tracked by 16 high-speed motion capture cameras with 120 Hz (Vicon® MX 13) to render an egocentric view of the virtual environment in the HMD in real-time. We used a NVIDIA Quadro FX 4600 graphics card with 768 MB RAM and a nVisor SX60 HMD with a field of view of 44° (horizontal) × 35° (vertical), a resolution of 1280 × 1024 pixels for each eye, and 100% overlap. The interpupillary distance was fixed at 6 cm. We adjusted the HMD fit and screen position for each participant. The overall setup provided important depth cues such as stereo vision, texture gradients, and motion parallax. During the whole experiment participants stood in front of the table and thus kept a constant physical body orientation.

The verbal shadowing task conducted during learning was a lexical-decision task (see 31 for details). Participants heard sound files via headphones and decided whether the sound was a German word or not by pressing mouse buttons. If no button press occurred within 1200 ms, a new trial started. This interval was shown to interfere with a concurrent spatial learning task.

### Procedure

After providing informed consent, participants came to the table and familiarized themselves with the joystick by pointing to locations within the laboratory. If they conducted a secondary task, it was explained to them and they trained for a couple of minutes while their baseline performance was measured. All participants were then equipped with the HMD, turned around once for disorientation, and started the *learning phase* in which they learned the object layout within the virtual environment from a single point of view. They were instructed to also look behind themselves to experience all possible views of the room. When participants claimed to know the layout, they proceeded to the learning test in which the array objects were removed from the room. An object name was displayed in the HMD and participants pointed with the joystick to the location where the object had been located before. They did so for all objects in pseudorandom order (i.e., not by rows or columns). Only when all pointing deviations were smaller than 15° did they proceed to the test phase; until then, they repeated the prior procedure. If used, verbal shadowing was provided throughout this procedure. Secondary task performance was only measured during learning and during baseline before that. During test pointing participants listened to verbal presentations, but were not required to react because this was too demanding.

In the *test phase* participants conducted an imagined and a visual pointing task in an order balanced between participants. In the imagined pointing task, participants read instructions on the HMD screen along the lines of “Imagine standing at A, facing B, point to C” where A, B, and C consisted of array objects. Following Kelly and McNamara [30] perspectives were evenly spaced around a full rotation in steps of 45°. Correct egocentric target direction was counterbalanced across imagined headings: in each imagined body orientation, participants pointed once to objects located ±45°, ±90°, and ±135° relative to the imagined body orientation (participants never had to point to their front or back). Visual pointing was identical, but participants saw the room from the location of object A and the overlying instruction read “You are at A, point to C”. Perspective had to be derived from the visual input in order to increase saliency of the surrounding room. Please note that both tasks relied on retrieved memory and were conducted from the same location. They thus could not rely on long-term memory vs. egocentrically updated environment as, for example, in [32]. Each task consisted of 48 trials (8 body orientations × 6 target directions) presented in a new random sequence for each participant and task.

In order to rule out interference between physical surrounding and imagined room orientation as an alternative explanation (as opposed to the visual virtual surrounding), participants faced a corner of the physical nonvisible room. Interference would have resulted in W-patterns with better performance at ±45° and ±135° instead of 0°, ±90° and 180°.

For pointing, participants pushed the joystick and pulled its trigger button. Latency consisted of the time taken between the onset of the instructions (and room) being presented and the button press. Pointing error consisted of the absolute deviation between pointing direction and correct direction.

### Design

The 2 × 2 × 2 × 8 mixed factorial design consisted of the within factors body orientation (eight levels) and pointing task (visual vs. imagined) and the counterbalanced between factors pointing task order (visual vs. imagined first) and verbal shadowing (yes vs. no) with 16 participants in each group.

### Data analysis

In order to control for outliers, we deleted values deviating more than 2 *SD* from a participant’s overall mean (ca. 4%). Data were submitted to an exploratory mixed model analysis with all factors. Compared to an ANOVA, mixed model analysis is less restrictive with regard to distribution assumptions [33]. Commonly accepted effect sizes for linear mixed models are not yet available. Thus we report partial eta square (*η*_*p*_^2^) derived from data aggregated per participant and the respective condition.

We used contrasts to describe V-, W-, and M-patterns centered on the learning orientation. Contrasts describe curve shapes within a single parameter thus instantiating a specific hypothesis and avoiding multiple testing in pairwise comparison between conditions [29,34]. A contrast weight of 0 in a perspective refers to average performance across perspectives as indicated by the black line in Figure 1 (right). Positive contrast weights predict higher latency or error rate than average. V-contrast weights were 2/1/0/−1/−2/−1/0/1, with 2 corresponding to 180° and −2 to 0° (i.e., the learning perspective); W-contrast weights were −1/1/−1/1/−1/1/−1/1, with 1 corresponding to ±45° and ±135°; and M-contrast weights were −2/0/2/0/−2/0/2/0, with −2 corresponding to 0° and 180° and 2 to ±90°. Contrasts were independent so experimental variations could enhance or reduce one contrast without at the same time necessarily enhancing or reducing another contrast. The sum of absolute contrast weights was 8, so contrast sizes could be compared with each other. For each participant, contrast weights were multiplied with the average performance in the respective perspective and summed (e.g., 2 × average in 180° + 1 × average in −135°, etc. for V-contrast). We compared the resulting contrast sizes in a 2 (verbal shadowing) × 2 (pointing task) × 2 (pointing task order) × 3 (contrast type) mixed model analysis. We predicted interactions of contrast type by experimental variation (shadowing or pointing task), which was followed by planned comparisons for individual contrasts. Adding participants’ sex to the analysis did not change any of the reported effects or reveal a main effect of sex. Therefore, only the pooled data are reported.

The contrasts described independent shape components. Overall shape might consist of multiple components adding up. In order to estimate which combination of components best described the pattern within a condition, we fitted the non-aggregated data in each condition with the following seven models: V, M, W, V+M, V+W, M+W, and V+M+W. We used the Akaike Information Criterion [35] to bias for fitting with more predictors and reported the best fitting model. Only positive predictors in line with the prediction of a hypothesis were considered.

## Results

### Verbal encoding

**Figure 2:**
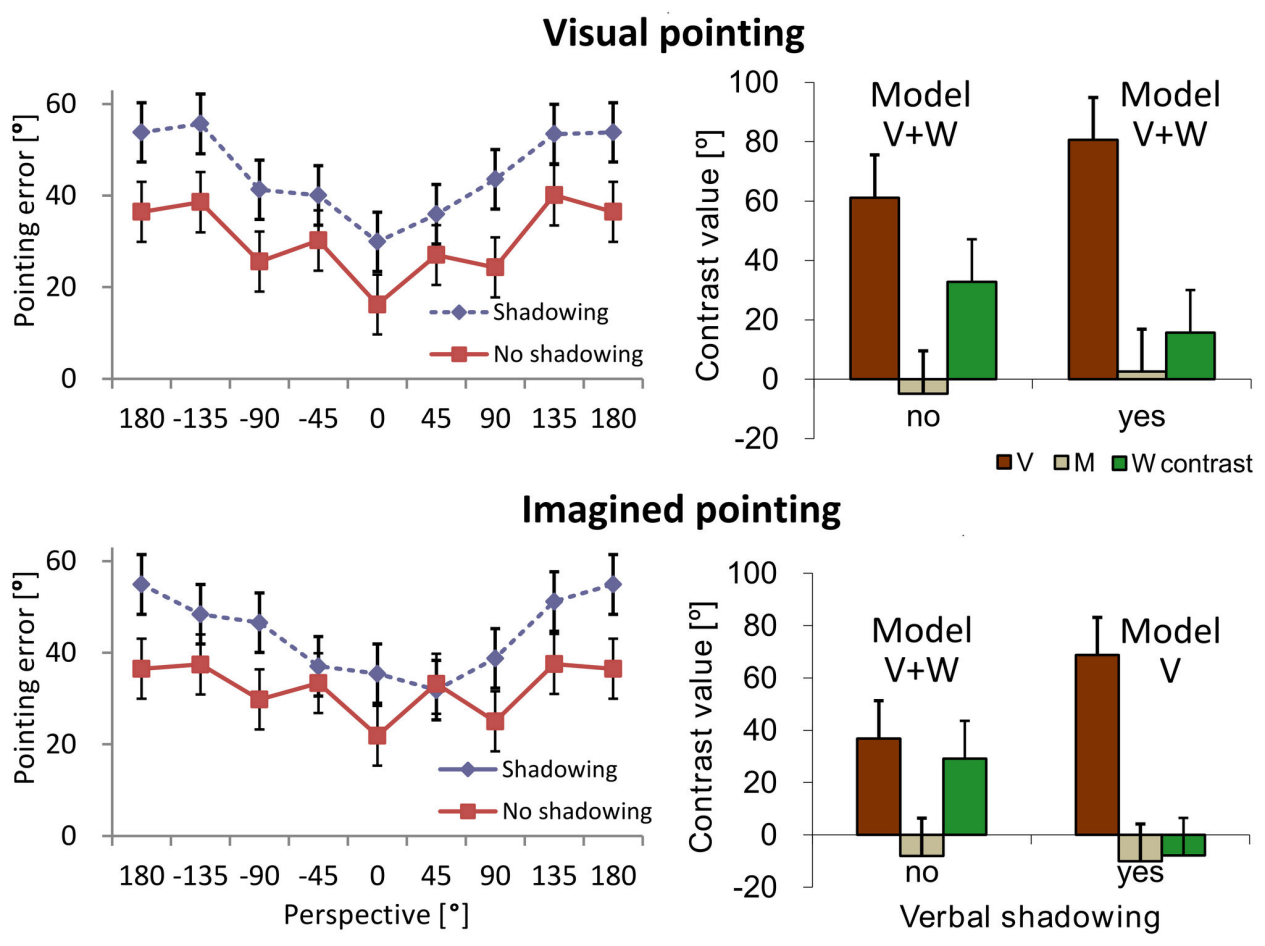
Pointing error. Left: Absolute pointing error as a function of perspective in the four conditions. Right: Corresponding contrasts. A contrast value of 0° would indicate that no V-, M-, or W-shape was present. Means and standard errors as estimated from the marginal means are shown. The best fitting model according the Akaike Information Criterion is shown.

As shown in Figure 2 (left), pointing error varied as a function of perspective, *F*(7, 2853) = 19.5, *p* < .001, *η* ^2^_*p*_ = .34. This variation was different between the verbal shadowing conditions as indicated by its interaction with perspective, *F*(7, 2853) = 2.22, *p* = .030, *η* ^2^_*p*_ = .06. The dashed and the continuous curves differed. The contrasts specified these pattern changes as shown in Figure 2 (right). As predicted, verbal shadowing interacted with the contrast type, *F*(2,140) = 3.73, *p* = .027, *η* ^2^_*p*_ = .05. Verbal shadowing reduced the W-contrast as compared to no verbal shadowing, *F*(1,28) = 8.40, *p* = .005, *η* ^2^_*p*_ = .27, the right W-contrasts were on average smaller than the left ones. No such difference was observed for the V- or M-contrasts, *F*(1,28) ≤ 1. Average contrast sizes differed, *F*(2,140) = 24.8, *p* < .001, *η* ^2^_*p*_ = .47. V-contrasts were larger than W-contrasts, *F*(1,84) = 20.6, *p* < .001, *η* ^2^_*p*_ = .28, which in turn were larger than M-contrasts, *F*(1,84) = 8.62, *p* = .004, *η* ^2^_*p*_ = .47.

Participants’ secondary task performance was both faster, *F*(1, 4521) = 29.5, *p* < .001, *η* ^2^_*p*_ = .32, and more accurate, *F*(1, 4704) = 376, *p* < .001, *η* ^2^_*p*_ = .93, during baseline (69%, *SE* = 2.8%, 974 ms, *SE* = 9.5 ms) than during learning the layout (35%, *SE* = 2.7%, 1016 ms, *SE* = 8.6 ms). Verbal shadowing interfered with layout learning. Both groups did not differ significantly in the time they spend learning the layout, *F*(1,28) < 1, which was 4.3 min (SE =1.9) for learning with verbal shadowing and 4.6 min (SE = 2.1) for learning without secondary task.

### Visual interference

**Figure 3:**
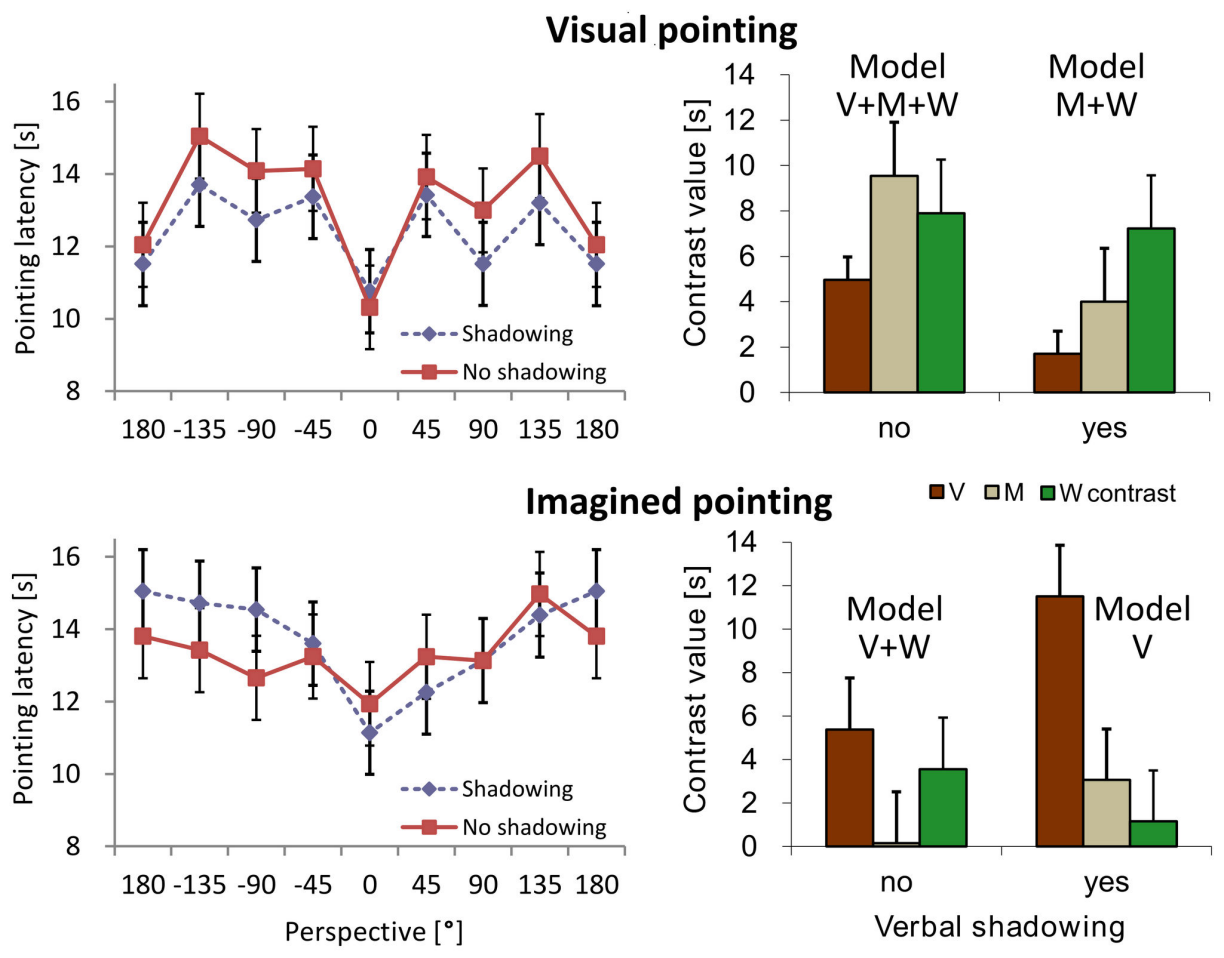
Pointing latency. Left: Pointing latency as a function of perspective in the four conditions. Right: corresponding contrasts.

As shown in Figure 3 (left), pointing latency varied as a function of perspective, *F*(7, 2854) = 11.3, *p* < .001, *η* ^2^_*p*_ = .24, and did so differently for the visual and the imagined pointing task as indicated in the interaction, *F*(7, 2854) = 3.21, *p* = .002, *η* ^2^_*p*_ = .09. The curves differed between panels. Figure 3 (right) shows how these differences were specified in the used contrasts. As predicted, pointing task and contrast type interacted, *F*(2,140) = 7.32, *p* = .001, *η* ^2^_*p*_ = .19. The W-shape was more prominent in visual pointing and thus visual interference –upper contrasts, than during imagined pointing without visual interference–lower contrasts, *F*(1,28) = 6.46, *p* = .017, *η* ^2^_*p*_ = .19. The same was found for the M-contrasts, *F*(1,28) = 6.19, *p* = .019, *η* ^2^_*p*_ = .18. V-contrast magnitudes were opposite with higher values in imagined than in visual pointing, *F*(1,28) = 4.10, *p* = .048, *η* ^2^_*p*_ = .12. Maybe there was a trade-off and higher V-contrasts compensated for the lower W- and M-contrasts.

Additional results in mean latency data (i.e., the level, not the pattern form) showed that visual pointing was, on average, quicker than imagined pointing, *F*(1, 2854) = 5.26, *p* = .022, *η* ^2^_*p*_ = .04. The interaction between task and order, *F*(7, 2854) = 11.3, *p* < .001, *η* ^2^_*p*_ = .08, indicated that participants pointed quicker in their second pointing task. And we found an interaction between shadowing and pointing task, *F*(1, 2854) = 7.01, *p* = .008, *η* ^2^_*p*_ = .05. Visual pointing was quicker after learning with shadowing than without shadowing, but not for imagined pointing. No other effects or interactions in errors or latencies attained significance.

### Modeling the pointing pattern

Verbal shadowing and imagined pointing both reduced the W-shape of the pointing pattern compared with no shadowing and visual pointing. When both came together, the W-pattern largely disappeared as indicated in the lower right contrasts of Figures 2 and 3. The data are best explained by the V-contrast only as suggested by the best fitting model. This does not rule out the possibility that W-patterns do still play a role. However, this role seems rather marginal compared with the V-shape. In all other conditions W-contrasts were relevant since they were part of the best fitting model.

## Discussion

Spatial memory is thought to be organized along experienced views or along orthogonal allocentric reference axes. Memory access from different perspectives yields V- and W-patterns, respectively. In the present experiment verbal shadowing reduced the W-pattern compared with no shadowing. This suggests that axes encoding was verbal in nature; for example, inter-object relations were described by rows and columns.

As an alternative explanation it was not the verbal nature of the secondary task that inhibited the formation of verbal memory traces. Axes encoding might have required extra resources during encoding which would have been blocked by any secondary task, verbal or not. If true, cognitive load from the secondary task did not just yield encoding towards one axis instead of two. This would have yielded an M-pattern during shadowing, but not without shadowing, which was not observed. We tested the 0°–180° axis. If participants used the −90° +90° axis instead, the M-contrast would have shown a strong negative value, which was not the case. It was also not the case that some participants used the 0°–180° axis and others the ±90° axis averaging out each other. Axes encoding theory explicitly states recording relative to axes’ endpoints [19]. The verbal secondary task thus clearly blocked encoding of spatial information relative to one or two axes.

Cognitive load may have reduced the encoded pattern not to one axis, but to one direction of an axis only, thus resulting in the V-pattern observed. This is a valid explanation of the present data. However, we think that the inhibition of verbal encoding is more plausible than general cognitive load. One reason for this it that participants had to pass a learn criterion. They continued learning with or without verbal shadowing until they were able to point to all locations with high accuracy. In such a situation general resource limitations from a secondary task could be compensated by extra learning time. We did not even observe longer learning times for learning with verbal shadowing which would have hinted in this direction. In case the resource limitations were specifically verbal, verbal memory traces were inhibited and participants had to rely more strongly on non-verbal memory such as visuospatial memory to pass the learning criterion. Such a switch in learning strategies does not necessarily require longer learning times for compensation, which were also not observed.

Verbal encoding also connects well with the literature, but less so with the cognitive load explanation. It is known that participants can form descriptions of object arrays and that subsequent pointing depends on which description (i.e., in which orientation) was previously formed [4]. Verbal encoding would state that participants memorized the description and used it for subsequent pointing. This also explains why descriptions and directions of best pointing coincide [20]. Furthermore, verbal encoding can predict *when* axes encoding and subsequent W-patterns will be observed, namely when verbal codes are used. For example, when learning object arrays arranged by rows and columns and giving judgments of relative directions afterwards [3,4,11–16]. Verbal codes may be suited for descriptions or judgments of relative directions, but less so for other tasks such as self-localizing. Verbal coding was shown to decline recognition performance [21]. In line with these considerations recognizing the very same object arrays does not show W patterns [2–4] presumably, because non-verbal memory rather than verbal memory was used for recognition. Capacity limitations through secondary tasks do not apply for these experiments and can therefore not explain why different patterns were observed. Verbal encoding can do so.

Taken together, verbal encoding connects well to the literature and may explain different outcomes in a wide range of experiments. Encoding relative to one orientation under load can explain results only in the present experiment. Furthermore, one could expect compensating resource limitations by extra learning time when learning to criterion as in the present experiment. Future experimentation might more clearly differentiate between these possibilities, for example, when learning an object array with a non-verbal secondary task or when influencing verbal coding by instruction rather than by a secondary task.

Both W and M-patterns were more prominent in visual compared with imagined pointing. We think that visual interference was the source for both effects. The room was rectangular and learning occurred while participants were oriented parallel to the long sides of the room. Pointing in the room from 0° and 180° was also parallel to the long walls. It was quicker than pointing from ±90°, which was aligned with the walls as well, but here the room elongated along the left–right body axis, not along front–back as during learning. When mentally superimposing the learned room onto the visible room higher similarity and thus less interference was present at 0° and 180° yielding the M-pattern observed. Wall alignment (0°, ±90°, and 180°) in general was better than wall misalignment, yielding the W-pattern as predicted. The more dissimilar the geometries were if mentally superimposed the stronger was the observed interference. So both the M and the W-pattern tie back to the specific rectangular shape of the room. Please note that the often observed performance increase in contra-aligned orientation of 180° [2,8,9] would have contributed to an M-pattern with or without interference. However, we did not find indications for this effect in the present experiment.

Visual interference assumes interference between the visible room geometry and the memorized room geometry. The visible room geometry was only virtual. Might the geometry of the physical room which was not visible during the experiment have played a role, too? We think we can exclude this possibility. Participants stood oblique to the walls of the physical room, facing a room corner. In case the non-visible physical room was represented and interfered with the visible virtual room during testing, an inverse W-pattern would have been observed with better performance at test orientations of ±45° and ±135° when the visible virtual room was aligned with the physical room. This was clearly not the case. An interesting opportunity for future research will be whether interference also occurs within physical rooms (see 27 for initial support).

The present experimentation can only be a first step indicating the possibility of visual interference. Future experiments will be needed to examine the exact circumstances under which such interference occurs. For example, in the present experiment attention was deliberately drawn to the visual geometry during testing, as participants had to rely on the room and the objects within for relocation. Will visual interference also occur when tested in a different room irrelevant for the task at hand and will visual interference vary with the similarity of such a novel room with the learning room? Present results suggest many routes for future experimentation.

The effects of verbal shadowing and visual interference were independent from each other. This suggests that W-pattern can originate from independent processes occurring during encoding as well as during retrieval. We speculate that they link also to different memory content namely inter-object relations within a (maybe verbal) axis code on the one hand and room geometry on the other hand. Two independent memory systems could be influenced independently by verbal shadowing as well as visual interference. Clearly, both experimental variations might have also affected one single spatial memory system. However, then interactions would seem plausible which were not observed.

Similarly, results do not seem to originate from choosing between mutual exclusive encoding strategies, for example, using either a (verbal) axis code or visuospatial coding. If this was the case reduced (verbal) axis encoding under shadowing should have yielded increased visuospatial coding resulting in stronger visual interference and vice versa. Again, such an interaction between verbal shadowing and visual interference was not observed.

Without verbal shadowing or a room visible, W-patterns were marginal, but V-patterns prevailed. In all other cases V- and W-patterns combined, in case of visual interference also with M-patterns. This suggests that V-patterns are the default – not only when recognizing objects or scenes, but also in judgments of relative direction. Experiments reporting W patterns only did not test for V-patterns, so V patterns might have been present as well [3,4,11–16]. Our results suggest that W-patterns only emerge when encouraged by (verbal) axis encoding or visual interference.

How do these results relate to allocentric and egocentric coding of spatial information? The experiment did not intend to examine the allocentric or egocentric nature of W- and V-patterns and can thus not make any suggestions by itself. W-patterns in relation to axes encoding have been typically associated with allocentric memory [1,19]. Verbal descriptions can clearly refer to non-egocentric reference directions and thus be allocentric [4]. The visual interference as described relies on room geometry. Memory of room geometry is also considered allocentric [28,36]. W-patterns thus seem to relate to allocentric memory. Contrary, V-patterns are typically centered on experienced egocentric orientations [2,4,5,7–9]. Egocentric views may thus underlie V-patterns in the present experiment as well.

Multiple combinations of memories and related processing seem possible to fully explain the present data 1 speculation striking to us is to assume verbal and visuospatial long-term memory. Verbal memory described inter-object relations along rows and columns and yielded W-patterns if constituted during learning. Visuospatial memory consisted of a 3D snapshot which included not only objects, but also the visible room geometry (cf. Figure 1). Retrieval was the quicker and accurate the more similar the retrieval and learning perspectives were yielding the V-pattern. In addition to the V-pattern, visual interference between snapshot and visible room geometry yielded the W-and the M-pattern with quicker responses the more similar both geometries were when superimposed. Similarity based retrieval processes in visuopatial memory thus accounted for V-patterns in general as well as M and W-patterns during visual interference. As both processes operated on visuospatial memory they traded-off with each other. V-patterns decreased with visual interference, M and W-patterns increased. Contrary, verbal shadowing operated on verbal memory and therefore did not influence visuospatial memory-based V-patterns. In summary, our speculation proposes that visuospatial memory yielded V-patterns in general as well as M and W-patterns during visual interference. Verbal memory independently added a W-pattern to that.

## Conclusions

Prior examinations into spatial memory differ on a fundamental aspect, namely whether memory access showed V-patterns or W-patterns. The present work gives first hints towards different cognitive processes underlying these patterns. If verbal memory organized along rows and columns was formed during learning, this may yield W-patterns in subsequent direction judgments. Alternatively, memorizing relative to spatial axes required extra cognitive capacity. Room shapes of a memorized room and the visible surrounding room may interfere with each other. This visual interference can yield W-patterns and as in the present case also an M-pattern (Verbal). axis encoding and visual interference seem to add onto processes yielding V-patterns and thus help in resolving different performance patterns in a wide range of experiments.
